# Agnostic Approvals in Oncology: Getting the Right Drug to the Right Patient with the Right Genomics

**DOI:** 10.3390/ph16040614

**Published:** 2023-04-19

**Authors:** Valentina Tateo, Paola Valeria Marchese, Veronica Mollica, Francesco Massari, Razelle Kurzrock, Jacob J. Adashek

**Affiliations:** 1 Medical Oncology, IRCCS Azienda Ospedaliero-Universitaria di Bologna, 40138 Bologna, Italy; 2 Department of Experimental, Diagnostic and Specialty Medicine, University of Bologna, 40127 Bologna, Italy; 3 MCW Cancer Center, Milwaukee, WI 53226, USA; 4 WIN Consortium, San Diego, CA 92093, USA; 5 Department of Oncology, University of Nebraska, Omaha, NE 68198, USA; 6 Department of Oncology, The Sidney Kimmel Comprehensive Cancer Center, The Johns Hopkins Hospital, Baltimore, MD 21287, USA

**Keywords:** agnostic treatments, biomarker-based, ALK, BRAF, FGFR, HER2, MSI, NTRK, RET, TMB

## Abstract

(1) Background: The oncology field has drastically changed with the advent of precision medicine, led by the discovery of druggable genes or immune targets assessed through next-generation sequencing. Biomarker-based treatments are increasingly emerging, and currently, six tissue-agnostic therapies are FDA-approved. (2) Methods: We performed a review of the literature and reported the trials that led to the approval of tissue-agnostic treatments and ongoing clinical trials currently investigating novel biomarker-based approaches. (3) Results: We discussed the approval of agnostic treatments: pembrolizumab and dostarlimab for MMRd/MSI-H, pembrolizumab for TMB-H, larotrectinib and entrectinib for NTRK-fusions, dabrafenib plus trametinib for BRAF V600E mutation, and selpercatinib for RET fusions. In addition, we reported novel clinical trials of biomarker-based approaches, including ALK, HER2, FGFR, and NRG1. (4) Conclusions: Precision medicine is constantly evolving, and with the improvement of diagnostic tools that allow a wider genomic definition of the tumor, tissue-agnostic targeted therapies are a promising treatment strategy tailored to the specific tumor genomic profile, leading to improved survival outcomes.

## 1. Introduction

### 1.1. Brief History of a Therapeutic Paradigm Change: The Revolution in Oncology

At the dawn of oncology, between the end of 1800 and the beginning of 1900, just a few Cancer Centers existed worldwide, and treatments consisted of surgery, x-rays, and the very first chemotherapeutic agents. Then, the decades of chemotherapy boom arrived, from 1940 to 1970, with a great expansion of available chemotherapeutic agents and combination schemes. Progress in cancer treatment was, from the beginning, always accompanied and actually driven by advances in diagnostic technologies, from the introduction of microscopy in pathology to the most modern molecular biology techniques [1,2]. Indeed, the first great step towards the new era of precision oncology was the discovery, in the late 1980s, of human epidermal growth factor-2 (HER2) overexpression or amplification in breast cancer, with the use of immunohistochemistry (IHC) first and then in situ hybridization (ISH) techniques. In the following few years, a humanized anti-human epidermal growth factor receptor 2 (HER2) antibody, trastuzumab, was engineered that received the first Food and Drug Administration (FDA) approval in 1998 [3,4]. The subsequent years were characterized by the development of several targeted therapies such as imatinib, a small molecule inhibitor of the BCR-ABL tyrosine kinase, in chronic myeloid leukemia (CML), and gefitinib, a small molecule inhibitor of epidermal growth factor receptor (EGFR), in non-small cell lung cancer (NSCLC). However, also with targeted therapies, most tumors went through tumor progression after a first variable period of response. Consequently, the research started to focus on resistance mechanisms to treatment (primary or acquired resistance, on-target or off-target, and so on) and new drugs or combinations to overcome resistance [4]. The second great step towards the revolution of the cancer treatment paradigm was the first human genome sequencing in 2001, but, even more, the advent of the next generation sequencing (NGS) techniques from ~2001, which eventually allowed today a rapid, economically sustainable, massive parallel DNA and RNA sequencing [4,5]. The increasing use of modern molecular biology technologies has enabled a deeper knowledge of the cancer molecular landscape, with the individuation of innumerable mutations, some of which are drivers that confer a selective growth advantage and others just bystanders/passengers. This has incredibly enriched, but also complicated, the world of oncology since the discrimination between driver and non-driver mutations is not always easy and immediate, and, in addition, not all driver mutations are targetable. In some cancer types, molecular subgroups based on the presence of specific driver mutations have been individuated, and specific targeted therapies have been developed, with consequent drastic improvements in prognosis [5,6,7,8,9]. Moreover, with the advent of immunotherapy, another powerful weapon against cancer has become available, achieving in some tumors satisfying and durable responses [10,11], as well as the potential interaction of amino acid residues in the priming of the immune system and response [12].

As a consequence of the huge expansion of precision oncology and immune oncology, two different waves in cancer research have caught on: combination therapies and molecular-specific/tumor-agnostic therapies. Since most cancers are not driven by a single molecular aberration, combination therapies composed of chemotherapy plus targeted therapy, chemotherapy plus immunotherapy, immunotherapy plus targeted therapy, or the combination of two immunotherapy agents have been investigated in order to increase the efficacy of the single treatments and overcome possible resistances, with several positive results in different cancers [13,14,15,16]. However, the advantages obtained with combination therapies, if not guided by the identification of specific mutations, are not always clearly imputable to a synergic effect of the combined drugs, but they could be due to a “larger coverage” of different subgroups responsive to different therapies. This could imply a “loss of precision” and consequent overtreatment of some subgroups of patients [5,17].

On the other hand, molecular-specific/tumor-agnostic therapies were born principally from two specific clinical needs: the finding of a tumor, a molecular aberration for which there was a targeted therapy already available for other tumor types; the finding of rare mutations/aberrations, potentially druggable, across different tumor types, including rare and ultra-rare-cancers. Due to the rarity of the conditions, both problems are characterized by a small sample size, preventing the design of a “traditional” single-histology trial. This situation entailed an increasing off-label use of molecularly targeted agents, with several promising case-reports publications, which, however, are characterized by an intrinsic bias since negative results of single cases are rarely published.

To overcome these problems, master protocols, in particular, histology-agnostic/aberration-specific basket trials [18,19,20] and N-of-1 trials [21,22,23], have been designed, largely adopted, and accepted by the drug regulatory authorities in the last years [4,5,6,19,24,25,26,27,28,29].

### 1.2. Agnostic Biomarkers and Therapies Co-Development

In the last few years, cancer treatment has been going through an astonishing renewal, with a radical change in the therapeutic paradigm, passing from tumor-specific/molecular-agnostic to molecular-specific/tumor-agnostic therapies. To meet the needs of the new oncology era, “untraditional” trial designs were implemented. Master protocols are studies composed of multiple subgroups/sub-studies, with patients affected by the same or different diseases, investigating the activity of one or multiple therapies.

Basket trials are a type of master protocol, which study the activity of a single drug in patients with different diseases, but sharing the same molecular aberration, divided into parallel sub-studies. The principle of tissue-agnostic inclusion is inspired by the classic phase I dose escalation design, which enrolled in a histology-independent manner to establish a recommended dose for the subsequent histology-specific phase [24,25,30]. Basket trials are based on the hypothesis that a biomarker could predict the response to a targeted therapy independently from tumor histology. This is a paramount point since the co-development of a predictive biomarker and a specific therapy is necessary for the successful development of a tissue-agnostic therapy. Basket trials allow one to investigate a drug in a molecular-specific small-size population and validate the predictive role of a biomarker. Moreover, this type of trial design enables to study of the effect of context since histology remains a fundamental variable: the disease-specific context of a targetable mutation may influence the activity of the targeted drug. Indeed, in different tumors, the weight of a molecular aberration could change due to the additional different aberrant pathways involved in the oncogenesis and resistance mechanisms. Besides that, other histology-specific factors could affect the response to a drug, first of all, the different tumor microenvironments that influence drug delivery and immunosurveillance, for example. Nevertheless, basket trials have some critical issues: due to the rarity of the investigated setting, they are normally characterized by a small dimension of the sample that normally prevents a randomized design; considering the single subgroups, the number of patients included could be extremely low, and so results could not be reliable; generally, they are phase I/II studies with a primary endpoint of activity and safety and not efficacy. Randomized controlled trials (RCT) should remain necessary for drug registrations, but in the case of exceptionally rare conditions or for targeted drugs that show remarkable early efficacy signals, a randomized trial may not be feasible or required for registration [6,30,31,32]. Basket trials are particularly useful for rare cancers or rare mutations, which are normally characterized by a driver mutation with low genomic complexity. Common cancers, instead, typically have an extremely complex molecular landscape characterized by several genomic, transcriptomic, and proteomic alterations, which influence the response to therapies.

The “N-of-1” is a trial design recently implemented in oncology with the aim of investigating an individualized molecular-driven treatment strategy. In this type of trial, each patient received a customized molecularly matched combination therapy that implies the necessity of an expert tumor molecular board to better interpret the molecular data of the single patient. Considering the increasing complexity of the personalized precision strategy in the new oncology era, it is also necessary to integrate with real-world data, large-scale registers, and master observational trials to allow a higher quality level of evidence for future approvals [5,21,22,23].

The aim of this review is to revisit the history of tissue-agnostic drug approval in oncology and illustrate the ongoing promising trials. This review will include the tissue-agnostic FDA approvals from 2017 to April 2023; we will not include partial approvals or germline approvals (i.e., belzutifan for germline).

## 2. Approved Tumor-Agnostic Treatments

Starting from 2017, with the first in oncology tissue-agnostic approval of pembrolizumab, many drugs were investigated and approved with a molecular-specific/tumor-agnostic indication.

Here we report the timetable of the tumor-agnostic drug approvals:

- 2017: pembrolizumab for patients with tumors deficient in mismatch repair (MMR) or with high microsatellite instability (MSI) (Section 2.1);
- 2018: larotrectinib for patients with neurotrophic receptor tyrosine kinase (NTRK) fusions-positive tumors (Section 2.3);
- 2019: entrectinib in patients with NTRK fusions-positive tumors (Section 2.3);
- 2020: pembrolizumab for patients affected by tumors with high tumor mutational burden (TMB) (Section 2.2);
- 2021: dostarlimab-gxly for patients with mismatch repair deficient tumors (Section 2.1);
- 2022: dabrafenib + trametinib in patients with BRAF V600E mutated tumors (Section 2.4);
- 2022: selpercatinib in patients with REarranged during Transfection (RET) fusion-positive tumors (Section 2.5).

In Table 1 are reported drugs that have already received tumor-agnostic FDA approval.

### 2.1. MMRd/MSI-H: Pembrolizumab and Dostarlimab

The MMR system (MSH2, MSH6, MLH1, and PMS2) recognizes and repairs mismatches of bases or erroneous insertion-deletion loops. Tumors with deficient mismatch repair (dMMR) accumulate frameshift mutations resulting in MSI, somatic hypermutated status, and finally increase in neoantigen formation, which are the targets of the immune system. Furthermore, MSI tumors frequently present an upregulation of immune checkpoint proteins and a rich lymphocyte infiltrate [33,34]. dMMR and high MSI (MSI-H) are predictive biomarkers of response to anti-programmed death 1 (PD1) blockade, as observed in clinical trials [35,36,37].

Pembrolizumab (KEYTRUDA) is a humanized IgG4 monoclonal antibody (mAb) that binds PD1, blocking the interaction with its ligands PD-L1 and PD-L2 and so preventing the PD-1-mediated inhibition of T-cell immune surveillance [38]. In May 2017, the FDA granted accelerated approval for pembrolizumab in adults and children affected by unresectable or metastatic solid tumors with MSI-H/dMMR, pretreated and without any valid alternative treatment option. The recommended dose is 200 mg every 3 weeks for adults and 2 mg/kg every 3 weeks for children [33,39]. That was the first tissue-agnostic approval in the history of oncology. The approval was based on the results of a pooled analysis of five single-arm trials: KEYNOTE-016, KEYNOTE-164, KEYNOTE-012, KEYNOTE-028, and KEYNOTE-158. The pooled analysis examined the effect of pembrolizumab on 149 patients with MSI-H/dMMR cancers enrolled in the five aforementioned studies. Colorectal cancer (CRC) was the most frequent tumor (90 patients). Pooled objective response rate (ORR) was 39.6%, with 11 (7.4%) complete responses (CR) and 48 (32.2%) partial responses (PR). ORR in CRC was 36% and 46% (33–59%; 95% CI) in the other cancers. At the time of the analysis, the median duration of the response (mDOR) was not reached. The DOR was ≥6 months in 78% of patients. Treatment-related adverse events (TRAEs) reported in patients with MSI-H/dMMR cancers were coherent with those already observed in previous trials with pembrolizumab [33,40]. The most frequent (reported in ≥20% of patients) were fatigue, musculoskeletal pain, rash, diarrhea, pyrexia, cough, decreased appetite, pruritus, dyspnea, constipation, pain, abdominal pain, nausea, and hypothyroidism. Immune-related TRAEs (irTRAEs), such as pneumonitis, colitis, hepatitis, endocrinopathies, and nephritis, were also observed [41]. The most recent publications about pembrolizumab have confirmed its durable and clinically significant benefit in patients with pretreated MSI-H/dMMR cancers, but also in first-line treatment for MSI-H/dMMR CRC, as observed from the results of the randomized KEYNOTE-177 trial [42,43,44,45].

Dostarlimab-glxy (TSR-042 or JEMPERLI) is a humanized IgG4 anti-PD1 mAb [46]. In August 2021, Dostarlimab received accelerated approval from the FDA for patients affected by recurrent or advanced solid tumors dMMR, in progression to a prior treatment or without satisfactory alternative treatment options, based on the results of the GARNET trial. The approval arrived a few months later; the FDA approved dostarlimab with the same indications but only in patients with endometrial cancers [47]. The GARNET trial (NCT02715284) is a multicenter, open-label, multi-cohort, phase I ongoing study designed to investigate the role of dostarlimab in patients with advanced solid tumors with poor therapeutic options. It consists of two parts: the first part evaluates increasing weight-based doses of the experimental drug with the aim to study safety, pharmacokinetics, and pharmacodynamics; the second one is composed of two subparts, the fixed-dose safety evaluation cohorts, and the expansions cohorts. The expansion cohorts are (1) MMRd/MSI-H endometrial cancers; (2) MMRp/MSS endometrial cancer; (3) non-small cell lung cancers (NSCLCs); (4) non-endometrial dMMR/MSI-H and POLE-mutated cancers; (5) high-grade serous, endometrioid, or clear cell ovarian, fallopian tube, or primary peritoneal cancer without BRCA mutations. The flat dose used in the expansion cohorts is 500 mg Q3W for the first four cycles and then 1000 mg Q6W for the subsequent cycles [48,49,50,51,52,53,54,55]. Here we report the principal results useful for the purposes of this review, that is to say, the results from cohorts of MMRd/MSI-H endometrial cancers and non-endometrial dMMR/MSI-H and POLE-mutated cancers. The most recent results were published on the occasion of the ASCO Annual Meeting 2022 and the ESMO Congress 2022. In the third interim analysis, 153 patients with MMRd/MSI-H endometrial cancers and 210 patients with non-endometrial dMMR/MSI-H and POLE-mutated cancers (56% CRC) were enrolled. The median follow-up was nearly 28 months for both cohorts. The ORR was 45.5% and 43.1% for the endometrial and non-endometrial cancers cohorts, respectively. The mDOR and the median overall survival (mOS) were not reached, while median progression-free-survival (mPFS) was 6 and 7.1 months for the endometrial and non-endometrial cancers cohorts, respectively [54]. In the overall dMMR population, ORR was 44%, with 13.1% of CR, 30.9% of PR, and 85.4% of ongoing responses. The disease control rate (DCR) was 58,4%, mDOR and mOS were not reached, while mPFS was 6.9 months [52]. The most frequent TRAEs were diarrhea, asthenia, nausea, and pruritus. Serious TRAEs occurred in 10% of patients. Patients that had to discontinue treatment due to TRAEs (alanine aminotransferase increased or pneumonitis) were 8.5% and 5.7% in the endometrial and non-endometrial cancers cohorts, respectively. With irTRAEs, principally hypothyroidism, alanine aminotransferase increased, and arthralgia was observed in 27% of patients. Two deaths attributed by investigators to study treatment were registered in the non-endometrial cancers cohort (one hepatic ischemia and one suicide) [52,54].

### 2.2. TMB-H: Pembrolizumab

TMB represents the number of somatic mutations in a tumor genome. Initially, whole-exome sequencing was used to assess TMB, so it evaluated only non-synonymous mutations presented in coding regions. Furthermore, germline mutations were excluded. More recently, a comprehensive genomic profiling (CGP) assay of 324 genes (FoundationOne CDx) has been approved by the FDA, and it also includes synonymous mutations and short insertions/deletions (indels) in intronic regions [56].

High TMB (TMB-H) is defined as the presence of ≥10 mutations/megabase (mut/Mb). High neoantigen production is associated with highly mutated tumors, and this is the rational basis for using immune checkpoint inhibitors (ICIs) in TMB-H tumors. Furthermore, the reason that MSI-H tumors respond to checkpoint inhibitors appears to be because MMR defects generate a high TMB, which translates into a high neoantigen load. The relationship between TMB and response to immunotherapy may be linear across cancers, and although the FDA used a cut-point for ≥10 mutations/mb for approving TMB as a pan-cancer marker, the optimal cut-point is still debated [56,57,58]. Of interest in this regard, in a study (90 patients; 19 tumor types) with the anti-PDL1 agent atezolizumab, the objective response rate was 38.1% versus 2.1% for patients with TMB ≥ 16 versus TMB ≥ 10 and 16 mutations/mb, suggesting that TMB is a robust biomarker for immunotherapy response, albeit at a higher cut off than 10 (e.g., perhaps 16 mutations/mb). In June 2020, the FDA accelerated the approval of pembrolizumab for patients with unresectable or metastatic solid tumors with TMB-H, pretreated or without valid alternative treatment options [59]. The approval was based on the results of the analysis of the cohorts of patients with TMB-H enrolled in the KEYNOTE-158 study [60]. KEYNOTE-158 is a multi-cohort, open-label, non-randomized, phase II ongoing study that enrolls patients with advanced solid tumors, pretreated with one or more lines of standard therapy, to receive pembrolizumab (200 mg) every 3 weeks. Patients are not selected for TMB status. TMB on tumor tissue is evaluated using the FoundationOne CDx assay, with a prespecified threshold for TMB-H of at least 10 mutations per megabase. The association between TMB status and the activity of pembrolizumab was evaluated in a prespecified analysis. At the time of this analysis, 805 patients of the safety population had an evaluable TMB, and of these, 105 (13%) had TMB-H. In the efficacy population, 790 patients had an evaluable TMB, and of these, 102 (13%) had TMB-H. After a median follow-up of 37.1 months, the ORR was 29% in the TMB-H group and 6% in the non-TMB-H group. mDOR had not been reached in the TMB-H group and was 33.1 months in the non-TMB-H group. mPFS and mOS were 2.1 months and 11.7 months in the TMB-H group and 2.1 months and 12.8 months in the non-TMB-H group, respectively. TRAEs occurred in 64% of patients, the most frequent of which were fatigue, asthenia, and hypothyroidism. Serious TRAEs were observed in 10% of patients, while treatment discontinuation occurred in 8% of patients. One death was assessed by the investigator to be treatment-related (pneumonia) [60].

### 2.3. NTRK-Fusions: Larotrectinib and Entrectinib

*NTRK1*, *NTRK2*, and *NTRK3* genes encoded for the tropomyosin receptor kinase (TRK) family (TRKA, TRKB, and TRKC), which are transmembrane receptors that bind the neurotrophins and activate downstream signaling cascades through phosphatidylinositol 3–kinase-protein kinase B–mammalian target of rapamycin (PI3K/AKT/mTOR), RAS/mitogen-activated protein kinase (MAPK)/extracellular signal-regulated kinase (ERK), and phospholipase C-gamma pathways. Abnormalities in the TRK pathway, principally *NTRK* gene fusions, are involved in cancer pathogenesis since they determine a constitutive activation of downstream pathways. *NTRK* fusions are rare events in the most common cancer histologies (prevalence of *NTRK* fusions < 5%) but are exceptionally frequent in some rare cancers such as infantile fibrosarcoma and secretory breast carcinoma (prevalence of *NTRK* fusions > 90%). In both cases, *NTRK* fusions have demonstrated an evident driver role in tumorigenesis and progression, making *NTRK* an optimum agnostic biomarker for targeted therapy with TRK inhibitors [61].

Larotrectinib (VITRAKVI or LOXO-101) is an adenosine triphosphate (ATP)-competitive and selective TRK inhibitor that has shown clinical activity in patients with TRK fusion-positive tumors [61]. In November 2018, larotrectinib received accelerated approval by the FDA for patients with metastatic or not operable solid tumors, pretreated or without any valid treatment option, harboring a fusion in the *NTRK* gene, and without a known acquired resistance mutation. The approval was based on the result of the analysis of the first 55 patients enrolled in three non-randomized clinical trials: (1) LOXO-TRK-14001, a phase I study; the first among all investigated larotrectinib in humans; (2) SCOUT, an ongoing phase I-II study of larotrectinib in children with solid tumors with *NTRK* fusion; (3) NAVIGATE, an ongoing phase II study of larotrectinib in adults and children with solid tumors with *NTRK* fusion [62,63]. Fifty-five patients with an age range from 4 months to 76 years and 17 different histological types of *NTRK* fusion-positive tumors received larotrectinib (100 mg) twice daily (if body surface area < 1 mq: 100 mg/mq twice daily). ORR determined by an independent radiology review committee was 75%, with 13% of CR (7 patients), 62% of PR (34 patients), 13% of SD (7 patients), 9% of PD (5 patients), and 4% not evaluated (2 patients). No correlation between response and tumor type, age, or TRK fusion type was observed. The 1-year PFS was 55%, with 71% of responses ongoing. The most frequent TRAEs were an increase in aminotransferase levels, dizziness, fatigue, nausea, and constipation. Of the 55 patients included in this analysis, no one discontinued treatment because of TRAEs, and no death related to treatment was registered [63]. A second pooled analysis was published more recently, including a larger population of 159 patients, with an age range from 1 month to 84 years. One hundred fifty-three patients were evaluable for response. The ORR was 79%, with 16% of CR and 63% of PR; 12% of patients had an SD and 6% PD. mDOR was 35.2 months, and, at 1 year, ongoing responses were 80%. mPFS was 28.3 months, with a 1-year PFS of 67%. mOS was 44.4 months, with a 1-year OS of 88%. Intracranial ORR was 75%. TRAEs were coherent with those reported in the first analysis [64].

Entrectinib (RXDX-101 or ROZLYTREK) is an inhibitor of the TRK family but also inhibits the proto-oncogene tyrosine-protein kinase ROS (ROS1) and the anaplastic lymphoma kinase (ALK) [61]. In August 2019, entrectinib received accelerated approval for patients with *NTRK* fusion-positive, solid, metastatic, or not surgical resectable tumors without a known acquired resistance mutation, pretreated, or without any valid alternative therapy. The approval was based on the results of three single-arm studies: ALKA-372-001, STARTRK-1, and STARTRK-2 [65,66]. A pooled analysis of 54 adult patients with *NTRK* fusion-positive metastatic or unresectable tumors enrolled in these three clinical trials were conducted [66]. Patients had 10 different tumor types and 19 distinct histologies, the most frequent sarcoma, NSCLC, and mammary analogue secretory carcinoma of the salivary gland (MASC). The recommended dose of entrectinib was 600 mg/day. ORR was 57% (with 7% of CR and 50% of PR. SD was the best response for 17% of patients. The response was observed in all tumor types and was independent of the type of *NTRK* fusion, except for *NTRK2*, which was present in only a patient who did not respond. mDOR was 10 months, while mPFS and mOS were 11 months and 21 months, respectively. The intracranial response rate was 55%, according to a blinded independent review. The majority of TRAEs were low-grade and reversible, but three serious TRAEs occurred in the *NTRK* fusion-positive population, in particular cognitive disorder, cerebellar ataxia, and dizziness. No deaths attributable to the treatment were reported [66]. The updated results of this pooled analysis included 121 patients affected by 14 different tumors and more than 30 distinct histologies and confirmed the significant clinical activity of entrectinib. ORR was 61.2%, while intracranial ORR was 63.6% [67].

### 2.4. BRAF V600E: Dabrafenib plus Trametinib

BRAF is a serine/threonine kinase of the RAF family and has a central role in the MAPK pathway. Mutations in *BRAF* have been observed in several cancers, with V600 mutations being the most common and best investigated. The prevalence of *BRAF* V600 mutations changes across different cancers. Tumors enriched for *BRAF* V600 mutations are thyroid cancer (prevalence > 40%), parathyroid cancer (prevalence > 30%), melanoma (prevalence > 20%), Langerhans cell histiocytosis (prevalence > 20%), and head and neck cancers (prevalence > 10%). CRC, gliomas, and gastrointestinal neuroendocrine cancers have a *BRAF* V600 mutation prevalence of 4–10%. Other common tumors in which *BRAF* V600 mutation has a significant prevalence are NSCLC, hepatobiliary cancer, ovarian cancer, and pancreatic cancer (prevalence < 4%). In other tumors, *BRAF* V600 mutations are extremely rare. Mutations of *BRAF* determine its constitutive activation and, consequently, downstream activation of MEK and ERK [68,69].

Dabrafenib (TAFINLAR) and trametinib (MEKINIST) are, respectively, BRAF and MEK inhibitors that demonstrated, first of all, significant clinical activity in *BRAF* V600 mutated melanoma [70]. In June 2022, the FDA granted accelerated approval to dabrafenib plus trametinib in patients with unresectable or metastatic solid tumors, pretreated or without a valid treatment option, harboring BRAFV600E mutations [71]. Patients with *BRAF* V600E mutated CRC are excluded from this indication because of the demonstrated resistance to BRAF inhibitors (instead, the combination of an anti-BRAF with cetuximab is approved in these patients) [71,72]. The recommended dose is dabrafenib (150 mg) twice daily and trametinib (2 mg) once daily. Approval was based on the data from 131 adult patients and 36 pediatric patients with *BRAF* V600 mutations enrolled in multi-cohort trials: NCI-MATCH subprotocol H, BRF117019 (ROAR trial), and part C and D of CTMT212X2101. The first study enrolled adult patients with several different tumors, the second enrolled adult patients with rare cancers, and the latter enrolled pediatric patients with low- (LGG) and high-grade gliomas (HGG). ORR in the 131 adult patients was 41%, while in the 36 pediatric patients, ORR was 25% [71]. Moreover, data were reinforced by the results of trials in melanoma and lung cancer in the first line, in which dabrafenib plus trametinib were formerly approved [70,73]. The NCI-MATCH subprotocol H enrolled 35 patients with different tumor types, except for melanoma, CRC, and thyroid cancers, harboring *BRAF* V600 mutations. Twenty-nine patients were included in the efficacy analysis. The ORR was 37.9%, with no CR observed, the mDOR was 25.1 months, and the DCR was 75.9%. mPFS was 11.4 months, while mOS was 28.6 months [74]. Results from the cohort of the gliomas (45 HGG and 13 LGG patients), biliary tract (43 patients), and anaplastic thyroid cancers (36 patients) from the phase II basket trial ROAR (BRF117019) were published. ORR in HGG was 33%, with 3 CR and 12 PR, while ORR in LGG was 69%, with 1 CR, 6 PR, and 2 minor responses. In biliary tract cancers, ORR was 51%, with no CR. In the anaplastic thyroid cancers, ORR was 56%, with 3 CR [75,76,77]. TRAEs in these trials were coherent with those reported with the use of dabrafenib plus trametinib in other indications, such as pyrexia, fatigue, nausea, rash, chills, headache, hemorrhage, cough, vomiting, constipation, diarrhea, myalgia, arthralgia, and edema [74,75,76,77].

### 2.5. RET Fusions: Selpercatinib

*RET* gene encodes for a transmembrane receptor tyrosine kinase that binds the ligand-coreceptor complex of glial cell line-derived neurotrophic factor (GDNF) family ligands (GFLs), with consequent downstream activation of signaling pathways such as RAS/ERK, RAS/MAPK, PI3K/AKT, and c-Jun N-terminal kinase (JNK). Germline gain of function mutations in *RET* are associated with multiple endocrine neoplasia 2 (MEN2) syndrome, while somatic *RET* mutations are observed in 60% of sporadic medullary thyroid carcinomas [78,79]. *RET* somatic mutations have been observed in several tumors. *RET* fusions are a type of somatic alteration due to chromosomal inversion or translocation that determines the formation of a chimeric *RET* protein with ligand-independent downstream signaling. *RET* fusions are more frequent in papillary thyroid carcinoma (PTC), with a reported frequency of 2.5–73%, and in NSCLC, with a reported frequency of 1–3%, but have also been reported in several other tumors at lower frequencies. The most common partner fusions are CDCC6 and NCOA4 in PTC and KIF5B in NSCLC [78].

Selpercatinib (RETEVMO or LOXO-292) is a highly selective, ATP-competitive, RET kinase inhibitor. In September 2022, selpercatinib received accelerated approval from the FDA for adult patients with locally advanced or metastatic solid tumors harboring *RET* gene fusion, pretreated or without any valid alternative therapy [80]. The recommended dose is 160 mg twice daily [81]. Approval was based on the results of the LIBRETTO-001 trial, in particular the data from 41 patients enrolled in the trial with tumors other than NSCLC and thyroid cancer, with the support of the data from 343 patients with NSCLC and thyroid cancer, enrolled in other cohorts of the same trial, in which selpercatinib was already label [79,82,83]. LIBRETTO-001 is a phase I/II, multi-cohort, ongoing trial that is investigating the activity of selpercatinib in patients with advanced solid tumors harboring *RET* gene alterations (fusions or mutations). Recently, data from 45 patients with *RET*-fusion-positive tumors other than thyroid cancer and NSCLC have been published. Enrolled patients had 14 different types of cancer, and almost all were pretreated (91%). The ORR in the evaluable efficacy population (41 patients) was 43.9%, with 16 PR and 2 CR. Responses were observed regardless of tumor type. The mDOR was 24.5 months, the mPFS was 13.2 months, while the mOS was 18 months. TRAEs were coherent with those previously reported, in particular, an increase in aminotransferases, dry mouth, diarrhea, ECG QT prolongation, and thrombocytopenia. The most common serious TRAEs were liver injury, fatigue, and hypersensitivity (each occurring in one of 45 patients).

**Table 1:** Drugs that have already received tumor-agnostic FDA approval.

Drug	Target	Date of Agnostic Approval	Indication	Evidence for Approval	Common Adverse Effects	References
Pembrolizumab	PD-1	23 May 2017	Patients with dMMR or MSI-H tumors	Pooled analysis on 149 patients enrolled across five single-arm studies. ORR: 39.6% (31.7–47.9%; 95% CI); DOR ≥ 6 months in 78% of patients.	pain in muscles, rash, diarrhea, fever, cough, decreased appetite, itching, shortness of breath, constipation, bones or joints and abdominal pain, nausea, and hypothyroidism	[38]
Larotrectinib	NTRK	26 November 2018	Patients with NTRK fusion-positive tumors	Pooled analysis on 55 patients enrolled across three single-arm studies. ORR: 75% (61–85%; 95% CI); 1-y PFS: 55%.	fatigue, nausea, dizziness, vomiting, increased AST, cough, increased ALT, constipation, and diarrhea	[63]
Entrectinib	NTRK	15 August 2019	Patients with NTRK fusion-positive tumors	Pooled analysis on 54 patients enrolled across three single-arm studies. ORR: 57% (43.2–70.8%; 95% CI); mDOR: 10 months (7.1-not estimable; 95% CI).	fatigue, constipation, dysgeusia, edema, dizziness, diarrhea, nausea, dysesthesia, dyspnea, myalgia, cognitive impairment, increased weight, cough, vomiting, pyrexia, arthralgia, and vision disorders	[66]
Pembrolizumab	PD-1	16 June 2020	Patients with TMB-H tumors	Subgroup prespecified analysis from a multi-cohort single-arm phase II study. ORR: 29% (21–39%; 95% CI); mDOR: not reached (range 22–34.8 months).	pain in muscles, rash, diarrhea, fever, cough, decreased appetite, itching, shortness of breath, constipation, bones or joints and stomach-area (abdominal) pain, nausea, and low levels of thyroid hormone	[60]
Dostarlimab	PD-1	17 August 2021	Patients with dMMR or MSI-H tumors	Prespecified interim analysis from a single-arm multi-cohort phase I study. ORR: 41.6% (34.9–48.6%; 95% CI); mDOR: 34.7 months (range 2.6–35.8).	fatigue/asthenia, anemia, rash, nausea, diarrhea, constipation, and vomiting	[46]
Dabrafenib + Trametinib	BRAF + MEK	22 June 2022	Patients with BRAFV600E mutated tumors	Pooled analysis on 167 patients (131 adults, 36 children) enrolled across three single-arm studies. ORR adults: 41% (33–50%; 95% CI); ORR children: 25% (12–42%; 95% CI).	pyrexia, fatigue, chills, peripheral edema, nausea, constipation, vomiting, diarrhea, rash, headache, hemorrhage, cough, myalgia, and arthralgia	[71]
Selpercatinib	RET	21 September 2022	Patients with RET fusion-positive tumors	Prespecified interim analysis from a multi-cohort single-arm phase I/II study. ORR: 43.9% (28.5–60.3%; 95% CI); mDOR: 24.5 months (9.2-not evaluable; 95% CI).	hypertension, prolonged QT interval, diarrhea, dyspnea, fatigue, abdominal pain, hemorrhage, headache, rash, constipation, nausea, vomiting, and edema	[82]

Abbreviations: PD-1, programmed cell death protein 1; NTRK, neurotrophic receptor tyrosine kinase; RET, REarranged during Transfection; dMMR, deficient mismatch repair; MSI-H, high microsatellite instability; ORR, overall response rate; DOR, duration of response; 1-y PFS, 1-year progression-free survival; mDOR, median duration of the response.

## 3. Agnostic Treatments Currently under Evaluation (Figure 1)

### 3.1. ALK

The anaplastic lymphoma kinase (*ALK*) gene encodes for a transmembrane tyrosine kinase receptor belonging to the superfamily of insulin receptors that was discovered for the first time in hematologic malignancies. The next *ALK*-related diseases discovered were inflammatory myofibroblastic tumor (IMT) [84,85] and the *EML4-ALK* rearrangement in NSCLC [86], which led to the detection of *ALK* alterations in other solid tumors such as neuroblastomas, rhabdomyosarcomas, and anaplastic thyroid tumors [87,88]. *ALK* gene can create a fusion protein with self-sustaining kinase activity through translocation with a partner gene, promoting tumor growth through the activation of various pathways, such as PI3K-AKT-mTOR, phospholipase Cy, Janus kinase–signal transducers and activators of transcription (JAK-STAT), and MAPK signaling [89,90]. Activation of the *ALK* gene can occur by rearrangements with partner genes, point mutations, or amplification.

**Figure 1:**
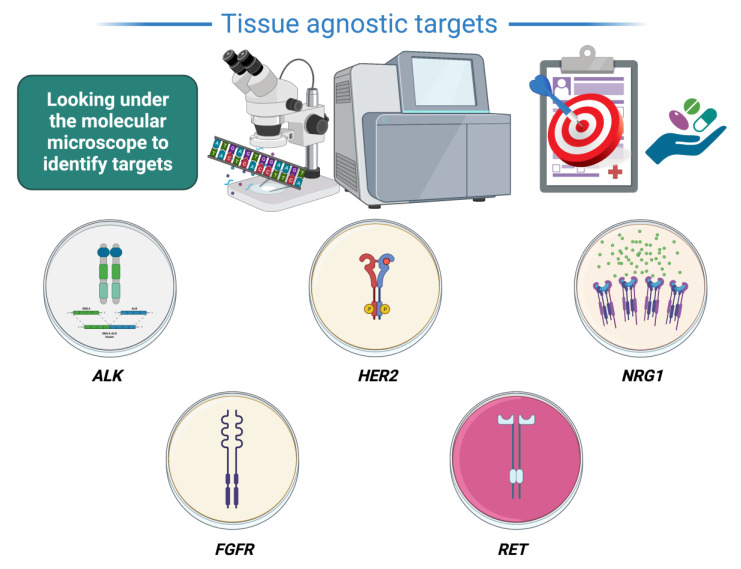
Ongoing clinical trials of tumor-agnostic treatments. Using the ‘molecular microscope’ or next-generation sequencing to identify novel targets that can be targeted with therapeutics.

Regarding the frequency of this alteration, a retrospective assessment of the Foundation Medicine database, including 114,200 clinical samples, showed that *ALK* fusions were present in 876 cases (0.8%) in total and, among them, the frequency of *ALK* fusions among NSCLC patients was 3.1%; conversely, in non-NSCLC tumors, the frequency was only 0.2% [91]. In this analysis, fusion partners varied in non-NSCLC and NSCLC tumors: in fact, most NSCLC patients presented an *EML4-ALK* fusion (83.5%), while these constituted the minority (30.9%) in non-NSCLC tumors. Its repercussion on response to *ALK*-directed therapies is unknown. *ALK*-fusions have been documented in CRC, biliary, and pancreatic tumors, showing often particular features [92,93]. In CRC and pancreatic cancer, the frequency of *ALK* rearrangements is much lower than NSCLC, accounting for <1% of colorectal cancer and <0.2% of pancreatic cancer. In metastatic CRC, *ALK* fusion is associated with a specific subtype, characterized by older age at diagnosis, *RAS* and *BRAF* wild-type status, microsatellite instability, significantly worse survival, and right-sided primary tumor location [93,94]. In gastric cancer, *ALK* overexpression was found in 8.4% of a series of patients with resected tumors and was associated with signet ring cell component and young age, as well as worse survival outcomes (DFS and OS) [95]. Pancreatic tumors showing *ALK* fusions are most frequently associated with *EML4* as a partner gene and appear to be associated with young age (<50 years), male gender, and wild-type *KRAS* status [92]. Since its discovery, *ALK* alterations have been a target for the development of therapies, and, given their ubiquitous presence, Hiroyuki Mano [96] has proposed the collective name *ALK*oma to refer to all those tumors that develop due to an alteration of the *ALK* gene.

The first-generation *ALK*-tyrosine kinase inhibitor (TKI) to be approved was crizotinib, which has been shown to have an important activity on *ALK*-positive solid tumors [91,97]. Alectinib is a second-generation *ALK* inhibitor that showed a higher response rate and lower toxicity than crizotinib in patients with metastatic *ALK*-rearranged NSCLC [98]. Moreover, the second-generation ALK inhibitors ceritinib and brigatinib and third-generation lorlatinib are employed in clinical practice for *ALK*-rearranged NSCLC [99,100]. Furthermore, phase II studies have demonstrated the activity of crizotinib in other tumor types, such as inflammatory myofibroblastic tumors (European Organisation for Research and Treatment of Cancer 90101 CREATE) [101].

With regards to alectinib, the level of evidence in *ALK*-positive solid tumors non-NSCLC in terms of efficacy and safety is weak and based only on case reports [102,103,104,105]. A recent case series by Takeyasu et al. [106] summarizes the effect of alectinib and crizotinib in patients with *ALK*-rearranged nonlung solid tumors (inflammatory myofibroblastic tumors, histiocytosis, histiocytic sarcoma, osteosarcoma, parotid adenocarcinoma): ORR was 85.7%, mPFS was 8.1 months, supporting the promising activity of ALK-TKIs in *ALK*-positive tumors. Even though, at the current moment, the role of this TKI remains established only for NSCLC [107,108], it is currently being investigated in cancer types different from NSCLC. An open-label, single-arm, phase II study of alectinib for patients with rare *ALK*-positive malignancies (JMA-IIA00364, TACKLE study) is ongoing and enrolls patients with *ALK* translocation, activating mutation, and overexpression, assessed through Foundation One next-generation sequencing profiling.

Even among gastrointestinal cancers, most of the data available on ALK-TKIs derives from case reports, which is not sufficient to support off-label use in many countries. Ambrosini et al. selected an international cohort of 13 patients with *ALK* fusion-positive gastrointestinal carcinomas, demonstrating the remarkable activity of several ALK inhibitors in heavily pretreated patients (ORR 41%, DCR 82%) [109]. Five patients (38%) received second-line ALK inhibitors, and two patients were still on second-line treatment at the time of data cut-off. Baskets trials are ongoing and could provide stronger prospective evidence on the role of ALK inhibitors in *ALK*-positive tumors.

The rarity of these alterations makes it difficult to explore possible scenarios that may occur based on different clinical and molecular characteristics, such as specific gene fusion partners or different *ALK*-targeting agents. Although rare, *ALK* translocations represent an important therapeutic target in many solid tumors beyond NSCLC. For example, rare cancers such as papillary renal cell carcinoma and salivary ductal carcinoma have also shown sensitivity to alectinib [103,110].

Patients with gastrointestinal malignancies harboring *ALK* translocations are at risk of being overlooked and excluded from personalized treatment that could significantly impact their survival. Therefore, further prospective studies are strongly needed to validate *ALK* rearrangement as a clinically useful therapeutic target.

### 3.2. HER2

HER2 is a transmembrane growth factor receptor, a member of the HER protein family, together with HER1 (EGFR), HER3, and HER4 [111]. *HER2* is an oncogene and exerts its role mainly due to gene overexpression, which increases its heterodimerization, favoring cellular transformation and tumorigenic growth [111]. Mutations occur mainly in the extracellular or kinase domain (90% of cases). The transmembrane and juxtamembrane domains are mutated in about 7% and 3% of cases, respectively [111]. To date, *HER2* has a key role in several solid tumors, and HER2-targeted therapies are FDA-approved for breast, gastric, and gastroesophageal cancers.

The first experimental studies of trastuzumab, the first anti-HER2 drug ever approved, were tested in the field of breast cancer (BC), where HER2 is expressed in about 15–20% of cases [112,113]. A subsequent HER2-targeting drug, ado-trastuzumab emtansine (T-DM1), received approval in 2013 for the treatment of trastuzumab-resistant metastatic BC [114]. In addition, two of the five antibody-drug conjugates (ADCs) currently approved by the FDA for solid tumors, trastuzumab deruxtecan (T-DXd) and sacituzumab govitecan (SG), are used for the treatment of advanced BC [115,116].

HER2 also plays a key role in metastatic gastric cancer, where anti-HER2 therapy is only approved for first-line treatment in HER2-positive metastatic gastric cancer, based on the phase III ToGA study, which demonstrated the benefit achieved by adding trastuzumab to standard platinum-based chemotherapy (mOS, 13.8 vs. 11.1 months; HR, 0.74) [117]. Studies of various combinations with different anti-HER2 molecules (e.g., dual blockade of HER2) have failed to demonstrate any survival benefit to date [118,119].

In the field of metastatic CRC, the prevalence of HER2 overexpression is higher in wild-type RAS/BRAF tumors (5–14%) and occurs more frequently in left-sided colon cancer [120]. The potential benefit of HER2-targeted therapy in wild-type RAS/BRAF mCRC has been evaluated in several clinical trials (e.g., HERACLES, MyPathway, TAPUR, Mountaneer) [121,122,123].

Furthermore, mutations in *HER2* have been detected in approximately 1–3% of NSCLCs, predominantly adenocarcinomas [124]. However, to date, there are no HER2-targeting drugs approved for this indication [125].

### 3.3. NRG1

The neuregulin 1 gene encodes the growth factor neuregulin 1 (NRG1). NRG1 contains an epidermal growth factor (EGF)-like domain, which binds to human tyrosine kinases of the ErbB/HER group of receptors, specifically ERBB3 and ERBB4. This binding aberrantly activates ErbB-mediated downstream signaling pathways that result in cell growth [126,127,128]. Although initially described in a breast cancer cell line [129], NRG1 fusions have recently been identified in many solid tumors with an extremely rare frequency (~0.1% to 0.3%) [130]. Two isoforms (α and β) of the EGF-like domain exist within the NRG1 ligand and confer differential binding affinities of NRG1 to members of the HER receptor family. The β isoform has a higher affinity for HER3 than the α isoform. The β-/α- isoform ratio could potentially modulate treatment response and resistance. Recently, an even rarer fusion of neuregulin-2 (NRG2+) was discovered [131], although its normal function remains to be fully elucidated. NRG1 fusions are present in many types of cancer, especially in a relatively high percentage of lung cancer, particularly invasive mucinous adenocarcinoma, which is one of the most aggressive types of lung cancer. Other NRG1-positive tumor types include pancreatic cancer, gallbladder cancer, renal cell carcinoma, bladder cancer, ovarian cancer, breast cancer, neuroendocrine tumor, sarcoma, and CRC.

Several clinical trials investigating the role of NRG1 as a target for agnostic treatment are ongoing. A phase II clinical trial is ongoing to investigate the efficacy of the pan-ERBB inhibitor afatinib in advanced NRG1-rearranged malignancies after progression with standard therapy [NCT04410653]. An open-label, single-arm, phase IV clinical trial is evaluating the efficacy of afatinib in the treatment of NRG1-fused locally advanced/metastatic NSCLC [NCT04814056]. Currently, afatinib is also being studied in two other studies [NCT02693535, NCT04410653]. Clinical trials are underway to test the efficacy of the drug seribantumab, a novel monoclonal antibody that binds to HER3 and inhibits NRG1-dependent activation and dimerization of HER2 [NCT01447706, NCT01151046, NCT00994123, NCT04790695, NCT04383210]. An open-label phase II study [NCT03805841] recruited patients with NSCLC (EGFR exon 20 insertion, HER2 activating mutations) and other solid tumors with NRG1/ERBB gene fusions and tested tarloxotinib bromide, a hypoxia-activated prodrug that generates a pan-HER TKI (tarloxotinib-effector). Zenocutuzumab (MCLA-128), a full-length IgG1 bispecific antibody that targets HER2 and HER3, is under investigation in phase I/II study in pretreated patients with solid tumors harboring an NRG1 fusion [NCT02912949]. Primary analysis on 85 patients from the eNRGytrial and 14 patients from the early access program with NRG1+ cancer was recently presented at ASCO 2022. Tumor types included NSCLC (*n* = 41), pancreas (n = 18), breast (*n* = 5 pts), colorectal cancer (*n* = 2), and cholangiocarcinoma (*n* = 3). Among the 71 evaluated patients, ORR was 34% with a median DOR of 9.1 months durable efficacy in pts with advanced NRG1+ cancer regardless of tumor histology [132].

### 3.4. FGFR

The fibroblast growth factor receptor (FGFR) family of proteins comprises four highly conserved transmembrane receptor tyrosine kinases (FGFR1-4). Natural activation of the receptor by fibroblast growth factor (FGF) ligands or its oncogenic alterations promotes cell proliferation, differentiation, morphogenesis and patterning, angiogenesis, and survival [133,134,135]. Alterations in this signaling pathway have been found in multiple types of human cancers. Notably, *FGFR2* fusions (associated with several partners, such as *BICC1*, *TACC3*, *CCDC6*, and *AHCYL1*) have been detected in approximately 10–20% of intrahepatic cholangiocarcinomas [136,137,138]. *FGFR1*-*3* fusions have been found in breast cancer, bladder cancer, glioblastoma, head and neck squamous cell carcinoma, low-grade glioma, lung adenocarcinoma, lung squamous cell carcinoma, ovarian cancer, prostate adenocarcinoma, and thyroid [139,140,141,142].

Given the role of FGFR signaling in tumorigenesis and progression, small molecule inhibitors targeting this signaling pathway have been developed (infigratinib, pemigatinib, erdafitinib) [143,144]. Positive results have been obtained from several clinical trials conducted in advanced pretreated cholangiocarcinoma patients with *FGFR2* fusion. Two phase II studies, one testing infigratinib, and one evaluating pemigatinib, demonstrated significant clinical efficacy in cholangiocarcinoma patients with *FGFR2* fusions [143,144]. Erdafitinib is approved for advanced or metastatic urothelial carcinoma with *FGFR3* mutation or *FGFR2/3* fusion [145] and also demonstrated efficacy in cholangiocarcinoma with *FGFR2* fusions [146].

The ongoing phase II RAGNAR study [NCT04083976] included a broad range of *FGFR*-altered tumor types, including tumors in which *FGFR* alterations are considered extremely rare (for example, pancreatic cancers). Thanks to its histological-agnostic design, the results of the RAGNAR study will help define the benefit of erdafitinib in several advanced solid tumors with *FGFR* alterations, including rare forms [147]. At the interim analysis data cut-off, responses were observed in 14 distinct tumor types, including gliomas, thoracic, gastrointestinal, gynecological, and rare tumors. The median values for DOR, PFS, and OS were 7.1 months, 5.2 months, and 10 months, respectively. DCR was 75.3%, and the clinical benefit rate (CBR) was 48.9%. The RAGNAR data showed, for the first time, the efficacy of erdafitinb in heavily pretreated patients and in rare and difficult-to-treat malignancies, including glioblastoma and pancreatic and salivary gland tumors [147]. However, the evidence is still very limited. Based on the data obtained so far, it seems promising to continue performing agnostic clinical trials to be able to best tailor treatment strategies to the specific patient.

### 3.5. RET

Pralsetinib (GAVRETO or BLU-667) is another RET inhibitor that showed meaningful clinical activity in the ARROW trial. This multi-cohort phase I/II trial enrolled patients with a solid tumor harboring a *RET* alteration (fusions or mutations). Results from the cohorts of patients with NSCLC and thyroid cancer provided in 2020 the FDA approval for these settings [148,149,150,151]. Recently, data from the tissue-agnostic cohort were published: the study enrolled 29 patients with 12 different *RET* fusion–positive solid tumors, other than NSCLC and thyroid cancer, pretreated or not a candidate for standard therapies. The ORR in the 23 patients of the efficacy analysis was 57%, with 3 CR and 10 PR and a DCR of 83%. Responses were observed independently from the histology or *RET* fusion partner. The mDOR was 11.7 months, the mPFS was 7.4 months, and the mOS was 13.6 months. The most common TRAEs were an aminotransferase increase, neutropenia, anemia, constipation, leucopenia, thrombocytopenia, asthenia, and hypertension. To date, pralsetinb has not been approved yet for tissue-agnostic indications by the FDA [152].

### 3.6. Combinations N-of-1

NGS has enabled the identification of potential targets for the development of new drugs aimed at neutralizing a specific mutation. As previously reported, there are many examples of drugs active against a specific mutation: the NTRK inhibitors larotrectinib and entrectinib in multiple solid tumors with *NTRK* fusions [63,66,153], the ROS1 inhibitors entrectinib and crizotinib in NSCLC with *ROS1* alteration [154,155], the FGFR inhibitor erdafitinib in *FGFR*-altered urothelial cancer [156]. However, most patients still do not benefit from single-agent targeted therapies, and most responders develop resistance. Several pieces of evidence suggest that optimized therapy may require an individualized combination approach (N-of-1 strategy) [22,23,157,158]. The first N-of-1 studies with customized combination included the I-PREDICT study [21,22,159] and the WINTHER study [23], with the latter also incorporating transcriptomics. In this setting, Molecular Tumor Board (MTB) face-to-face meetings have been shown to successfully facilitate the interpretation of multiple test modalities, including tissue NGS, cfDNA, mRNA, and IHC. With this approach, patients are more likely to receive a more personalized therapy showing significantly better clinical outcomes [155].

Therefore, given that monotherapies are likely to be inefficient and may not provide lasting results in some patients, collegial discussion remains a pivotal step. Some oncological drugs that lack efficacy as single agents could produce durable responses when combined with other compounds with a different mechanism of action [159,160]. For example, some studies show the modest efficacy of single-agent EGFR inhibitors in gastric cancer [158] and single-agent CDK (cyclin-dependent kinase) inhibitors in breast cancer [161,162]. Although, combinations such as those of CDK inhibitors with antiestrogens are effective, indicating that drug combinations are needed to enhance the activity [163].

Oncology is moving more and more towards biomarker-driven treatments. In addition, there is the improvement of genomic technologies, through the study of transcriptomes and proteomes, both in the preclinical phase and through related studies in clinical trials [155]. The need for better patient selection through the definition of specific biomarkers will be used to predict response to combination therapies [164]. Analysis of genomic alterations in cancer of unknown primary (CUP) patients studied by NGS of tissue or blood-derived cfDNA revealed that 3.6% of tumors had a genetic MSI-H repair defect/mismatch and 23% had TMB ≥ 10 mutations/mb, which are both FDA approved tissue-agnostic genomic biomarkers for immunotherapy. Moreover, 30.9% of CUPs were PD-L1 positive by IHC, also an FDA-approved biomarker for checkpoint blockade [165]. Furthermore, the simultaneous occurrence of several genomic alterations within an immune portfolio highlights the need to always obtain an NGS analysis to allow for the selection of an individualized combination treatment. Consistent with this observation, the degree to which CUP was matched to a tailored treatment combination (a post hoc calculated match score equivalent to a number of targeted alterations/total number of deleterious alterations) was the only factor independently associated with better outcomes. Considering the complex molecular profile of CUP, selecting treatments with a higher degree of affinity to molecular alterations correlate with better outcomes. Emerging data suggest that the administration of targeted drugs to unselected patients is associated with a paltry ORR < 5% [21,166]. Studies involving unselected patients are occasionally successful by accident alone, so it is becoming increasingly clear that choosing an increasingly unscreened treatment will benefit patients and facilitate advances in cancer research overall. Further clinical investigations are needed to validate these findings, as well as to determine if there are additional parameters that can predict the utility of precision therapies.

## 4. Conclusions

**Figure 2:**
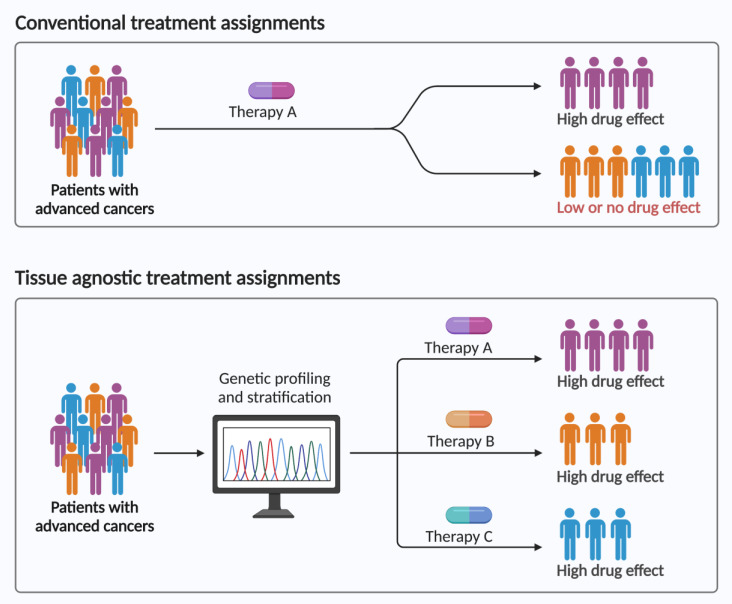
Conventional versus tissue-agnostic trials. Conventional trials often designate all patients with a specific advanced cancer to a drug that may or may not match their cancer genomic alterations. This often leads to a benefit in a select group of patients but not for everyone. Tissue-agnostic basket trials based on genomic alterations specific to a patient’s cancer may lead to more optimal therapy designations and better treatment effects.

There currently are seven tissue-agnostic FDA approvals for patients with advanced cancers. These allow patients access to novel therapies that they would otherwise likely not be able to access. It also allows for patients with rare cancers that may not ever have a clinical trial dedicated to their cancer or specific genomic alteration, including access to such drugs. The current landscape of clinical trial design and the ability to accrue patients to genomic-driven basket trials will likely aid in the advancement of additional tissue-agnostic approvals (Figure 2). Conventional trials include patients who derive benefit from therapies, but often retrospective post hoc analysis gives insight into why certain patients may have benefited more than others. Similar analyses for patients on tissue-agnostic trials will likely help elucidate potential resistance mechanisms, which in turn will further the field of precision oncology. Patients with advanced cancers want to live and want an opportunity to try therapies that may benefit them. Tissue-agnostic approvals give these patients those opportunities, and more approvals will likely lead to more lives saved or prolonged for patients.

## Data Availability

Not applicable.
